# Syndrome of inappropriate secretion of thyroid-stimulating hormone in a subject with galactorrhea and menstrual disorder and undergoing infertility treatment: Case report

**DOI:** 10.1097/MD.0000000000028414

**Published:** 2021-12-30

**Authors:** Hideaki Kaneto, Shinji Kamei, Fuminori Tatsumi, Masashi Shimoda, Tomohiko Kimura, Atsushi Obata, Takatoshi Anno, Shuhei Nakanishi, Kohei Kaku, Tomoatsu Mune

**Affiliations:** a Department of Diabetes, Endocrinology and Metabolism, Kawasaki Medical School, Japan; b Department of Diabetic Medicine, Kurashiki Central Hospital, Japan; c Department of General Internal Medicine 1, Kawasaki Medical School, Japan; d Kawasaki Medical School General Medical Center, Japan.

**Keywords:** hyperthyroidism, infertilitiy treatment, pituitary adenoma, syndrome of inappropriate secretion of thyroid-stimulating hormone, TSH-secreting pituitary adenoma

## Abstract

**Rationale:**

Syndrome of inappropriate secretion of thyroid-stimulating hormone (SITSH) is a rare cause of hyperthyroidism. Thyroid-stimulating hormone (TSH) levels are usually normal or high, and triiodothyronine (FT_3_) and free thyroxine (FT_4_) levels are usually high in subjects with SITSH.

**Patient concern:**

A 37-year-old woman had experienced galactorrhea and menstrual disorder for a couple of years before. She had undergone infertility treatment in 1 year before, hyperthyroidism was detected and she was referred to our institution.

**Diagnosis:**

She was suspected of having SITSH and was hospitalized at our institution for further examination. The data on admission were as follows: FT_3_, 4.62 pg/mL; FT_4_, 1.86 ng/dL; TSH, 2.55 μIU/mL. Although both FT_3_ and FT_4_ levels were high, TSH levels were not suppressed, which is compatible with SITSH. In addition, in brain contrast-enhanced magnetic resonance imaging, nodular lesions were observed in the pituitary gland with a diameter of approximately 10 mm. In the thyrotropin-releasing hormone load test, TSH did not increase at all, which was also compatible with TSH-secreting pituitary adenoma. In the octreotide load test, the TSH levels were suppressed. Based on these findings, we diagnosed this subject as SITSH.

**Interventions:**

Hardy surgery was performed after the final diagnosis. In TSH staining of the resected pituitary adenoma, many TSH-producing cells were observed. These findings further confirmed the diagnosis of pituitary adenoma producing TSH.

**Outcomes:**

Approximately 2 months after the operation, TSH, FT_3_, and FT_4_ levels were normalized. Approximately 3 months after the operation, she became pregnant without any difficulty.

**Lessons:**

We should consider the possibility of SITSH in subjects with galactorrhea, menstrual disorders, or infertility. In addition, we should recognize that it is very important to repeatedly examine thyroid function in subjects with galactorrhea, menstrual disorder, or infertility.

## 1 Introduction

Syndrome of inappropriate secretion of thyroid-stimulating hormone (SITSH) is a rare cause of hyperthyroidism and accounts for 1% to 2% of all pituitary adenomas. Thyroid-stimulating hormone (TSH) levels are usually normal or high, and triiodothyronine (FT_3_) and free thyroxine (FT_4_) levels are usually high in subjects with SITSH. Thyrotoxicosis and swelling of the thyroid are often observed, while exophthalmos is rarely observed in patients with SITSH. Headache is often observed in patients with SITSH due to enlargement of the pituitary adenoma. In addition, some cases of SITSH can have galactorrhea or menstrual disorder due to increased levels of prolactin (PRL) and/or decreased levels of luteinizing hormone (LH) or follicle stimulating hormone (FSH), as well as increased levels of TSH.[^1^,^2^,^3^,^4^,^5^,^6^] When we fail to recognize the presence of SITSH, thyroid ablation may be needed to further expand the pituitary tumor volume. The first therapeutic approach to SITSH is pituitary neurosurgery. The medical treatment of SITSH is mainly administration of somatostatin analogs such as octreotide, which are effective in reducing TSH levels. SITSH is induced by resistance to thyroid hormone (RTH) in addition to TSH-secreting pituitary adenoma (TSHoma), and it is important to perform a differential diagnosis of TSHoma and RTH.[^7^,^8^,^9^,^10^]

Here, we present a patient with SITSH due to TSHoma who had galactorrhea, menstrual disorders, and headache for a long period of time and underwent infertility treatment in the Department of Obstetrics and Gynecology, but who became pregnant smoothly after the operation of TSHoma and finally gave birth safely without any difficulty.

## 2 Case presentation

A 37-year-old woman had experienced galactorrhea, menstrual disorders, and headache for several years. In addition, she had undergone infertility treatment in the Department of Obstetrics and Gynecology since about 1 year before. It is well known that thyroid dysfunction could influence fertility, thyroid function was checked several times, thyroid ultrasonography was performed, and there was no abnormality in such examinations for a couple of years. However, hyperthyroidism was detected once, and the patient was referred to our institution. The data in our institution were as follows: FT_3_, 4.90 pg/mL; FT_4_, 2.06 ng/dL; TSH, 2.06 μIU/mL. Since FT_3_ and FT_4_ levels were high but TSH levels were not suppressed, she was suspected of having SITSH and was hospitalized in our institution for further examination.

On admission, his height, body weight, and body mass index were 167.3 cm, 63.4 kg, and 22.6 kg/m^2^, respectively. Blood pressure, heart rate, and body temperature were 120/68 mm Hg, 52 beats/min, and 36.2°C, respectively. The data on admission were as follows: FT_3_, 4.62 pg/mL; FT_4_, 1.86 ng/dL; TSH, 2.55 μIU/mL. Although both FT_3_ and FT_4_ levels were high, TSH levels were not suppressed, which was compatible with TSHoma. Thyroid autoimmune antibodies were all negative: antithyroid peroxidase antibody, 9.9 IU/mL; antithyroglobulin antibody, <10.0 IU/mL; thyrotropin receptor antibody, 1.0 IU/L; TSH antibody, 106%. PRL showed a normal upper limit (18.6 ng/mL), and LH and FSH were both lower than the reference range: LH, 1.8 mIU/mL; FSH, 2.8 mIU/mL. Growth hormone (GH) and insulin-like growth factor-1 levels were within normal ranges. Peripheral blood and electrolyte levels were within the normal range. Liver and renal function, diabetes, and lipid markers were all within normal ranges.

**Figure 1:**
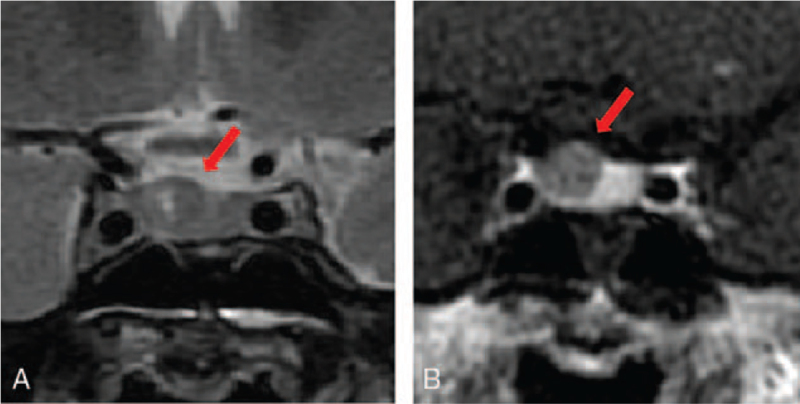
(A) In brain contrast-enhanced magnetic resonance imaging (MRI), nodular lesion was observed in the pituitary gland and its diameter was approximately 10 mm. (B) In dynamic study, the nodule lesion was slightly contrasted, but contrast effect was weaker compared to the adjacent pituitary gland.

**Figure 2:**
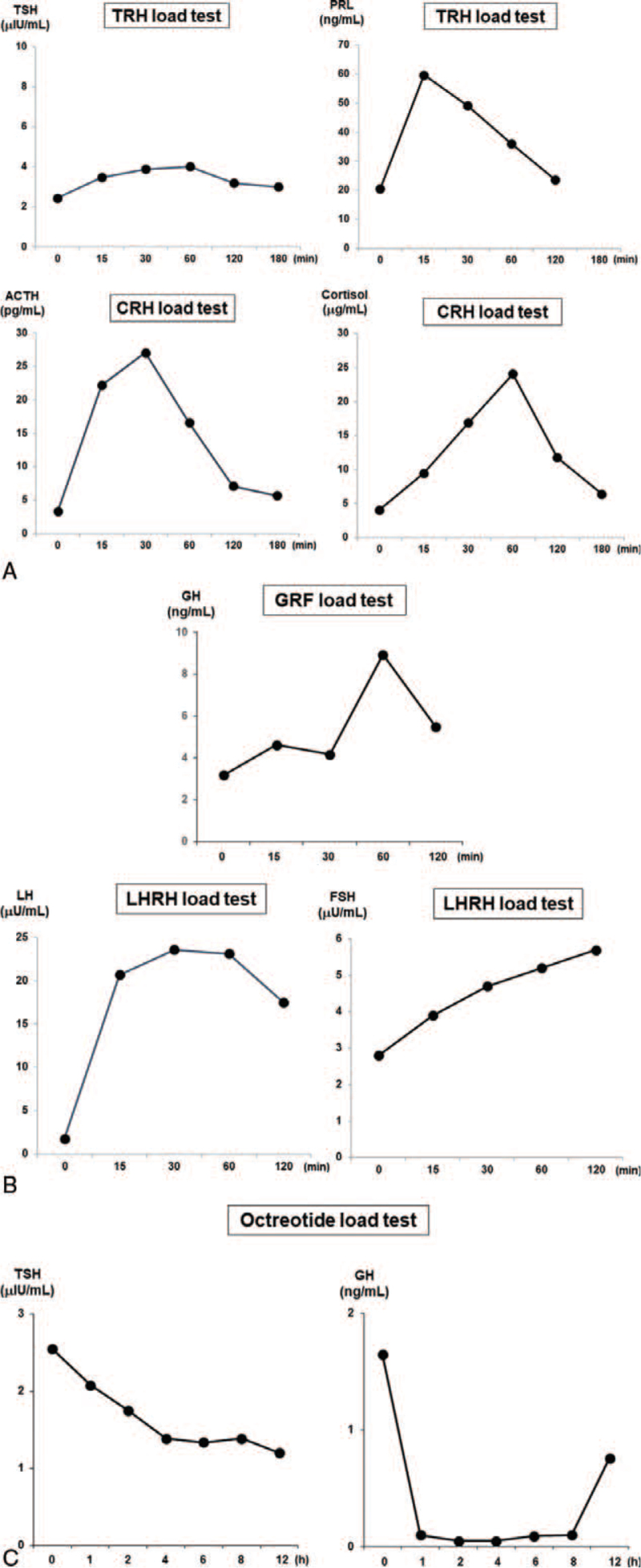
In TRH load test, TSH was not increased at all which was compatible with TSHoma. In contrast, PRL was increased normally. In CRH load test, both ACTH and cortisol levels were increased normally. In LHRH load test, LH and FSH levels were both increased. In GRF load test, GH level was increased normally. In octreotide load test, TSH level was suppressed. ACTH = adrenocorticotropic, CRH = corticotrophin-releasing hormone, FSH = follicle stimulating hormone, GH = growth hormone, GRF = growth hormone-releasing, LH = luteinizing hormone, LHRH = luteinizing hormone-releasing hormone, PRL = prolactin, TRH = thyrotropin-releasing hormone, TSH = thyroid-stimulating hormone, TSHoma = TSH-secreting pituitary adenoma.

In addition, in brain contrast-enhanced magnetic resonance imaging (MRI), nodular lesions were detected in the pituitary gland with a diameter of approximately 10 mm (Fig. 1A). In the dynamic study, the nodule lesion was slightly contrasted, but the contrast effect was weaker than that of the adjacent pituitary gland (Fig. 1B). On thyroid ultrasonography, there was no abnormality: no swelling of the thyroid, no increase in blood flow, and no neoplastic lesion in the thyroid. In the thyrotropin-releasing hormone (TRH) load test with 0.5 mg of TRH, TSH did not increase and subsequently FT_3_ and FT_4_ levels did not increase, which was compatible with TSHoma (Fig. 2). In contrast, PRL increased normally upon TRH stimulation. In the corticotrophin-releasing hormone load test with 100 μg of corticotrophin-releasing hormone, both adrenocorticotropic hormone and cortisol levels increased normally. In the luteinizing hormone-releasing hormone load test with 0.1 mg), both LH and FSH levels increased normally. In the growth hormone-releasing factor load test with 100 μg of growth hormone-releasing factor, the GH level increased normally. In octreotide load test with 100 μg of octreotide, TSH level was suppressed (Fig. 2), and FT_3_ and FT_4_ levels were both suppressed 12 hours after octreotide load as follows: FT_3_, from 4.62 pg/mL to 3.74 pg/mL; FT_4_, from 1.65 ng/mL to 1.50 ng/mL.

In addition, it is important to perform a differential diagnosis of TSHoma and RTH in patients with SITSH. In the case of TSHoma, imaging is positive, and the TSH response is absent in the TRH load test. In this subject, in brain contrast-enhanced MRI, nodular lesions were clearly detected in the pituitary gland (Fig. 1), and TSH levels were not increased at all in the TRH load test (Fig. 2). In addition, while TSH secretion is usually suppressed in the octreotide load test in subjects with TSHoma, TSH secretion was clearly suppressed by octreotide in this subject. Furthermore, while subjects with RTH often have a family history, this subject did not have a family history. Based on these findings, we ruled out the possibility of RTH and finally diagnosed this subject as SITSH due to TSHoma.

**Figure 3:**
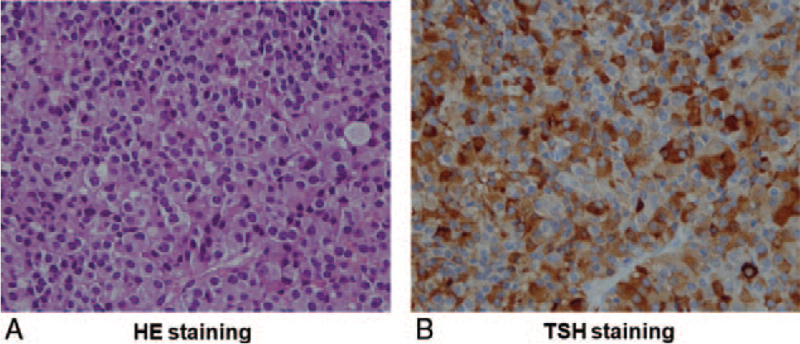
(A) In HE staining of the resected pituitary adenoma, solid proliferation of chromophobe cells was observed. (B) In TSH staining, many TSH-producing cells were observed in the resected pituitary adenoma. HE = hematoxylin and eosin, TSH = thyroid-stimulating hormone.

After the final diagnosis, a Hardy operation was performed. The dissected pituitary adenoma was yellow in color, and its size was 10 mm × 10 mm × 12 mm. Hematoxylin and eosin staining of the resected pituitary adenoma revealed solid proliferation of chromophobe cells (Fig. 3A). In TSH staining, many TSH-producing cells were observed in the resected pituitary adenoma (Fig. 3B). These findings further confirmed the diagnosis of SITSH. About 2 months after the operation, TSH, FT_3_, and FT_4_ levels were decreased as follows: TSH, 0.47 μIU/mL; FT_3_, 2.64 pg/mL; FT_4_, 0.71 ng/dL. After the operation, she did not need any anterior pituitary hormone replacement, although slight pituitary hypothyroidism was transiently observed, as described above. Approximately 3 months after the operation, she became pregnant without any difficulty. She was 38 years old at that time, and therefore, she had some risk due to relatively late maternity. Nonetheless, she finally gave birth safely without any difficulty, and her baby's body weight was 3060 g. In addition, in follow-up brain contrast-enhanced MRI taken about 1 year later, there was no abnormality in the pituitary gland; there were no findings indicating the occurrence of TSHoma.

## 3 Discussion and conclusions

Here, we present a subject with SITSH due to TSHoma who had galactorrhea and menstrual disorders for a long time and underwent infertility treatment in the Department of Obstetrics and Gynecology. About 3 months after the operation of TSHoma, she became pregnant and gave birth safely without any difficulty. It is known that the miscarriage rate is quite high in subjects with high TSH levels, although infertility and miscarriage due to hypothyroidism can usually be prevented by appropriate treatment. Therefore, it is possible that this subject would have continued to suffer from infertility and might have experienced miscarriage without the operation.

It was reported that clinical manifestations in patients with TSHoma were as follows^[4]^: previous thyroidectomy, 29%; severe thyrotoxicosis, 21%; goiter, 93%; thyroid nodule,7 0%; macroadenoma, 76%; visual field defect, 35%; headache, 21%; menstrual disorder, 33%; galactorrehea, 28%; and acromegaly, 16%. In general, signs and symptoms of hyperthyroidism are frequently observed in subjects with TSHoma, thus causing thyrotoxicosis, goiter, thyroid nodules, visual field defects, and loss of vision. In particular, goiter is very often observed even in previously thyroidectomized subjects, because thyroid residue can grow again as a consequence of TSH hyperstimulation. In this subject, however, signs and symptoms of hyperthyroidism were not observed at all; there were no thyrotoxicosis, goiter, thyroid nodule, visual field defect, or loss of vision. In addition, the patient did not have a history of thyroidectomy. These points made it difficult to diagnose TSHoma. It was also reported that most subjects had a history of thyroid dysfunction and were frequently misdiagnosed with Graves’ disease. It has been reported that approximately 30% of patients had inappropriate thyroidectomy or radioiodine thyroid ablation.[^1^,^8^,^11^] The patient did not have a history of thyroid dysfunction, and autoantibodies for Graves disease (thyrotropin receptor antibody and TSH antibody) were negative. Menstrual disorders are observed in almost all women with TSHoma plus prolactinoma, one-third of those with TSHoma, and central hypogonadism, delayed puberty, and decreased libido are often observed in males with TSHomas. In this subject, menstrual disorder and galactorrehea were observed, which was compatible with TSHoma. In addition, it is highly controversial that the diagnosis of TSHoma is often delayed in subjects with autoimmune hypothyroidism plus TSHoma.[^12^,^13^,^14^] We should bear in mind that inadequate suppression of TSH during thyroid hormone replacement suggests the presence of TSHoma.

TSHoma secretes large amounts of TSH inappropriately, thereby secreting large amounts of thyroid hormones. It is likely that increased TSH and thyroid hormones forcibly enhance metabolism in the whole body and lead to disturbed fertility. In addition, PRL and/or GH secretion is also enhanced in some subjects with TSHoma.[^1^,^2^,^3^,^4^,^5^,^6^] Since TSH-producing thyrotrophs, PRL-secreting lactrophs, and GH-producing somatotrophs are all derived from Pit-producing cells, we assume that such commonality of their origins is associated with the increased secretion of PRL and/or GH in some subjects with TSHoma. In this subject, PRL showed a normal upper limit, which might have, at least in part, facilitated the disturbance of fertility. In addition, in this subject, levels of LH and FSH, both of which are important hormones for pregnancy, were lower than the reference range, which might also have facilitated fertility disturbance. Therefore, we assume that various hormonal changes such as increased TSH and thyroid hormones, relatively increased PRL, and decreased LH and FSH levels were associated with galactorrhea, menstrual disorders, and infertility in this subject.

Taken together, we should bear in mind the possibility of SITSH in subjects with galactorrhea, menstrual disorders, and infertility because delayed diagnosis of SITSH leads to infertility or miscarriage. Therefore, we should recognize once more that it is very important to repeatedly examine thyroid function in subjects with galactorrhea, menstrual disorder or infertility.

## Author contributions

**Investigation:** Fuminori Tatsumi, Masashi Shimoda, Tomohiko Kimura, Atsushi Obata, Takatoshi Anno, Shuhei Nakanishi, Kohei Kaku, Tomoatsu Mune.

**Writing – original draft:** Shinji Kamei.

**Writing – review & editing:** Hideaki Kaneto.
